# Mechanical unloading is accompanied by reverse metabolic remodelling in the failing heart: Identification of a novel citraconate‐mediated pathway

**DOI:** 10.1002/ejhf.3704

**Published:** 2025-06-04

**Authors:** David M. Kaye, Xiao Suo Wang, Yen Chin Koay, Mengbo Li, Bailey McIntosh, Yann Huey Ng, Michael Rahman, Yiyang Cao, Francine Z. Marques, Cassandra Malecki, Shane Nanayakkara, Justin Mariani, Bing Wang, Sean Lal, Giovanni Guglielmi, John F. O'Sullivan

**Affiliations:** ^1^ Heart Failure Research Group, Baker Heart and Diabetes Institute Melbourne VIC Australia; ^2^ Department of Cardiology Alfred Hospital Melbourne VIC Australia; ^3^ Monash‐Alfred‐Baker Centre for Cardiovascular Research, Monash University Melbourne VIC Australia; ^4^ Cardiometabolic‐Medicine Laboratory, The University of Sydney Camperdown NSW Australia; ^5^ Charles Perkins Centre, The University of Sydney Camperdown NSW Australia; ^6^ School of Medical Sciences, Faculty of Medicine and Health, The University of Sydney Camperdown NSW Australia; ^7^ Bioinformatics Division The Walter and Eliza Hall Institute of Medical Research Parkville VIC Australia; ^8^ Department of Medical Biology The University of Melbourne Parkville VIC Australia; ^9^ Hypertension Research Laboratory, School of Biological Sciences, Faculty of Science, Monash University Melbourne VIC Australia; ^10^ Department of Biomedical Engineering The University of Melbourne Parkville VIC Australia; ^11^ School of Mathematics, University of Birmingham Birmingham UK; ^12^ Department of Cardiology Royal Prince Alfred Hospital Sydney NSW Australia; ^13^ Faculty of Medicine, TU Dresden Dresden Germany

**Keywords:** Myocardial metabolism, HFrEF, LVAD, Remodelling, Metabolomics, Lipidomics

## Abstract

**Aims:**

Although functional recovery of the failing heart with left ventricular assist device (LVAD) unloading can occur, the underpinning mechanism is unclear. We aimed to characterize the effect of myocardial biochemical effect of LVAD support *in vivo* and *in vitro*.

**Methods and results:**

We performed targeted metabolomics and lipidomics on transcardiac (arterial and coronary sinus) blood samples collected from healthy volunteers (*n* = 13), patients with end‐stage heart failure with reduced ejection fraction (HFrEF, *n* = 20), and LVAD‐supported HFrEF patients (*n* = 18). Complementary biochemical studies in myocardial tissue samples from healthy donor, HFrEF and LVAD patients, and cardiomyoblasts were performed. Myocardial uptake of intermediates in purine, nucleotide, and tricarboxylic acid (TCA) cycle pathways was depressed in HFrEF patients, with recovery in LVAD patients. Glucose uptake was suppressed in HFrEF but restored in LVAD. Metabolite changes suggestive of impaired fatty acid oxidation were present in HFrEF but not in LVAD. We found that the metabolite citraconate was significantly released by HFrEF hearts compared to controls and this was corroborated, in separate patients, by increased levels of citraconate in HFrEF myocardium but not in LVAD. Whilst citraconate increased succinate deydrogenase (SDH) activity in cardiomyoblasts, its isomer itaconate suppressed SDH activity. SDH activity was maintained in HFrEF myocardium but was diminished in LVAD myocardium.

**Conclusions:**

We report, for the first time, the *in‐vivo* biochemical effects of LVAD unloading in the human heart. Our data identify citraconate as a potentially important regulator of the TCA cycle in the failing heart.

## Introduction

Despite improvements in the pharmacological management of heart failure (HF) with reduced ejection fraction (HFrEF), outcomes continue to be limited, particularly in more advanced disease states. HFrEF progression is underpinned by ventricular remodelling, a collective process which incorporates deleterious changes in cardiac structure and function driven by exposure to mechanical, neurohormonal and biochemical stressors.^1^ For patients with the most advanced forms of HFrEF, left ventricular assist device (LVAD) also provides an opportunity for aggressive unloading and reverse remodelling of the heart.^2^ In a small proportion of cases, the extent of reverse remodelling has been such that the magnitude of functional recovery has been sufficient to allow LVAD explantation.^2^

The paradigm of LVAD unloading of the failing heart and reverse remodelling provides an opportunity to explore the potential mechanisms that contribute to HFrEF progression and, thereby, which could be addressed for intervention. Amongst key targets, improvement in contractile performance depends on adequate cardiac energetics. In contrast to non‐failing myocardium, in which the majority of adenosine triphosphate (ATP) is generated from the oxidation of fatty acids, the failing heart has been demonstrated to be more dependent on glycolysis, as recently reviewed.^3^ In particular, the tricarboxylic acid (TCA) cycle plays a critical role in the generation of ATP. Overflow of TCA cycle intermediates is known to cause myocardial injury,^4^ and potential drivers of myocardial fibrosis, although the effect of mechanical unloading is uncertain. Furthermore, whilst the determinants of TCA flux under normal conditions and in the setting of ischaemia are known, this is less clear in HFrEF and LVAD patients. Whilst some insights into the metabolic effects of LVAD‐mediated unloading have been gained, most studies have been performed *ex‐situ* in tissues obtained at LVAD implant or transplant and thus impacted upon by the effects of cardioplegia and fasting.

We therefore aimed to investigate the cardiac metabolic and energetic effect of LVAD support in comparison to HFrEF patients and healthy controls by obtaining arterial and coronary sinus blood samples with subsequent targeted metabolomic analysis supported by studies in isolated left ventricular tissue samples and cultured cardiac cells.

## Methods

### Study population and catheterization protocol

The study included 20 HFrEF patients, 18 LVAD patients, and 13 healthy control subjects. The control subject samples have been previously reported on^5^ and are provided to contrast with the HFrEF metabolomic data. HFrEF and LVAD patients were recruited from the Advanced Heart Failure Service of the Alfred Hospital, Melbourne. All patients were haemodynamically stable at the time of study. Healthy volunteers were recruited from the general community with no history of significant comorbidities. LVAD patients were recruited at the time of a routine haemodynamic evaluation study and HFrEF patients were studied during assessment for heart transplantation and were not receiving inotropic support. The protocol for haemodynamic evaluation and transcardiac (arterial and coronary sinus) blood sampling has been previously described.^5^ The study was approved by the Alfred Hospital Research and Ethics Committee, and all participants gave written informed consent.

### Cardiomyoblast experiments

Cardiomyoblast cell line H9C2 derived from embryonic rat heart tissue (ATCC CRL‐1446) were cultured in Dulbecco's Modified Eagles Medium (Gibco) supplemented with 10% v/v foetal bovine serum (Thermo Fisher Scientific) and 1% w/v penicillin–streptomycin (Gibco) at 37°C with a humidified atmosphere of 5% CO_2_. The H9C2 cardiomyoblasts were seeded in 6‐well plates at a density of 5 × 10^5^ cells/well. When the cardiomyoblasts reached approximately 70–80% confluency, they were treated with 5 mM citraconic acid (Sigma) or 5 mM itaconic acid (Sigma) for 6, 12 or 24 h.

### Left ventricular tissue samples

Left ventricular samples were obtained from explanted hearts of end‐stage HF patients undergoing heart transplantation surgery or at the time of LVAD implantation for severe HF, and from healthy/non‐pathological donors whose hearts could not be viably used for heart transplantation due to logistical and compatibility limitations. Samples were stored in liquid nitrogen. The methods of procurement, storage and use of donated human myocardium were approved by the Human Research Ethics Committee at The University of Sydney (USYD 2021/122).

### Metabolomic and lipidomic analyses of plasma and myocardial tissue

Metabolites were extracted from frozen heart tissues as previously described.^6^ Targeted metabolite profiling used in this study was established using reference standards for each individual metabolite to determine mass spectrometry (MS) multiple reaction‐monitoring transitions, declustering potentials, and collision energies and chromatographic retention time, as described previously.^7^, ^8^ For both HILIC and AMIDE analysis, a liquid chromatography–MS/MS system composed of a Sciex Qtrap 6500 Plus mass spectrometer (AB Sciex, Framingham, MA, USA), connected to a Shimadzu Nexera LC‐40C series system (Shimadzu Corp., Kyoto, Japan) was used, as previously described.^7^, ^9^ Lipidomic profiling in this study used a Thermo Scientific™ Vanquish™ UHPLC system and Thermo Scientific™ Q Exactive™ HF‐X Hybrid Quadrupole‐Orbitrap™ mass spectrometer as described previously.^10^, ^11^ Lipid Search 4.1 software was used to align and process each acquired raw file's search results, as well as to identify the lipid species present in the heart extracts.^12^

The convention we used in the text for clarity is the fold change (FC) between efflux/coronary sinus and influx/arterial blood, with FC <1 meaning metabolites and lipids were lower in coronary sinus versus arterial blood and extracted by the heart, and FC >1 meaning release from the heart.

### Statistical analysis

Clinical parameters are summarized with mean (± standard deviation) of the samples. Quantitative clinical data, such as body mass index and age, were compared between cohorts using the Welch's *t*‐test. To investigate the heterogeneity of subgroups of individuals between cohorts, for example, sex unbalance or individuals with hypertension between two cohorts, we employed the test of proportion. Metabolites and lipids measured in coronary sinus and artery were normalized using a hierarchical method to remove unwanted variation.^13^ The tissue data and the cardiomyoblasts were normalized using EigenMS.^14^

The differential abundance analysis was performed using two‐tailed limma moderated *t*‐test^15^ on log2‐scaled abundance, corrected by age, biological sex, and body mass index of the individual; the individual was accounted as a random effect for transcardiac gradient data. The *p*‐values were then adjusted using the false discovery rate procedure (*p*_fdr_). Pathways analysis was conducted using CAMERA^16^ and KEGG^17^ on the transcardiac gradient analysis; similarly, analysis of the lipids and metabolite classes was conducted using LipidSearch classification^18^ and Human Metabolome Database (HMDB) chemical classes,^19^ respectively. The succinate deydrogenase (SDH) activity was tested via two‐tailed non‐parametric pooled bootstrap *t*‐test.^20^ The Uniform Manifold Approximation and Projection (UMAP) was applied on kNN (k = 10) imputed data.^21^

## Results

The demographic, clinical, echocardiographic and haemodynamic features of the study subjects are presented in online supplementary *Table* *S1*. As expected, the stable LVAD patients had a better NYHA status and more favourable haemodynamics than HFrEF patients. There were some differences in HF therapy between groups (HFrEF vs. LVAD), beta‐blockade (70% vs. 39%, *p* = 0.054), and mineralocorticoid receptor antagonist (75% vs. 28%, *p* = 0.004). At the time of commencement, sodium–glucose cotransporter 2 inhibitor therapy was not included in the international HF guidelines at the start of the study, therefore we did not include patients on therapy to avoid potential confounding.

### Myocardial metabolite and lipid changes in heart failure with reduced ejection fraction

**Figure 1:**
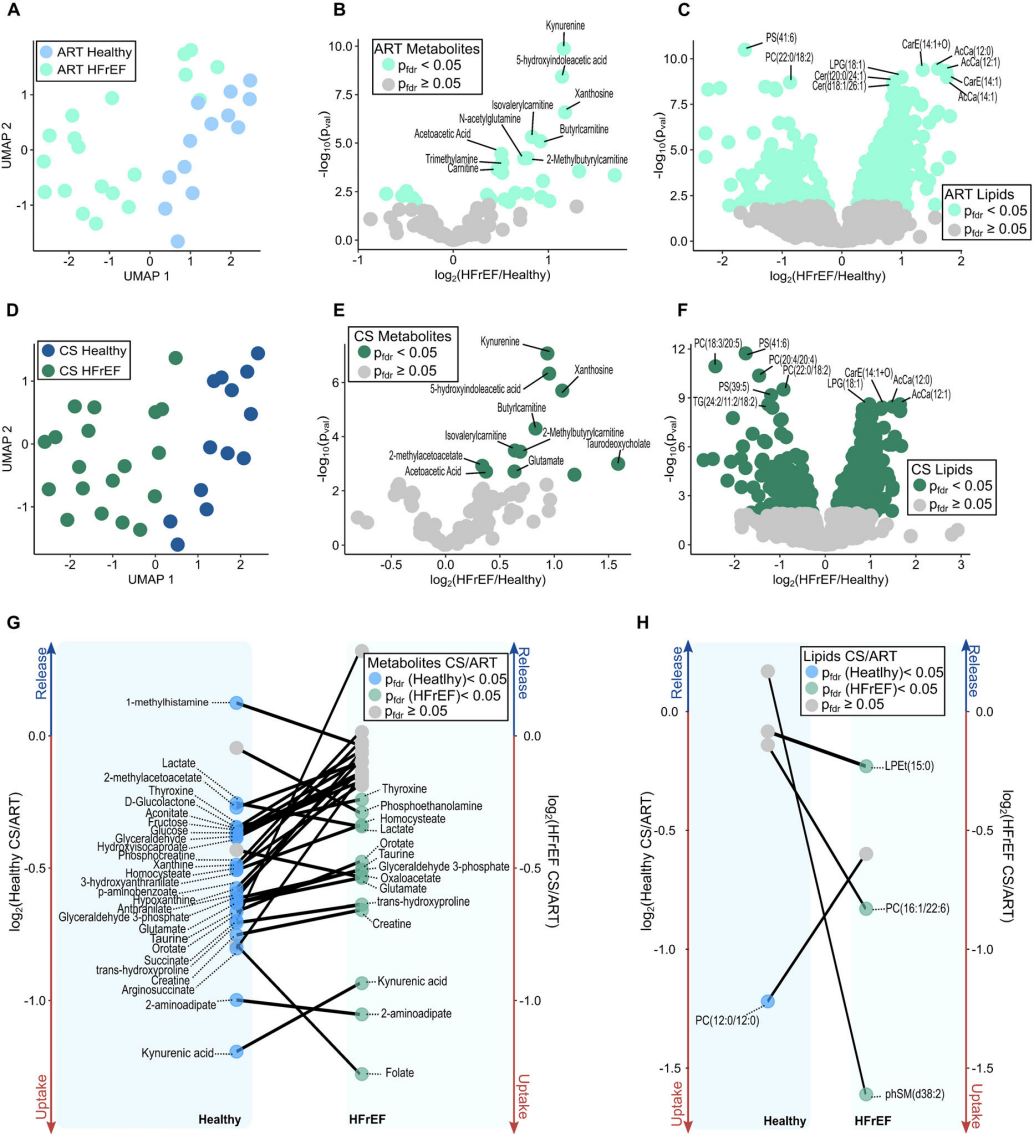
(*A*) Uniform Manifold Approximation and Projection (UMAP) of healthy and heart failure with reduced ejection fraction (HFrEF) artery samples. (*B*) Arterial (ART) metabolites detected compared in HFrEF and healthy individuals. (*C*) ART lipids compared in HFrEF and healthy individuals. (*D*) UMAP of healthy and HFrEF coronary sinus (CS) samples. (*E*) CS lipids compared in HFrEF and healthy individuals. (*F*) In the volcano plot, CS lipids detected compared in HFrEF and healthy individuals. (*G*) The parallel plot counterposes released/extracted metabolites (*p*_fdr_ < 0.05) in healthy with those in HFrEF. (*H*) The parallel plot counterposes released/extracted lipids (*p*_fdr_ < 0.05) in healthy with those in HFrEF.

As shown in *Figure* *1A*, the combined arterial metabolomic and lipidomic profile of HFrEF patients was distinct from that observed in healthy controls when analysed by UMAP. Amongst several metabolites, plasma levels of kynurenine (FC = 2.23, *p*_fdr_ < 0.001), 5‐hydroxyindoleacetic acid (5‐HIAA) (FC = 2.21, *p*_fdr_ < 0.001) xanthosine (FC = 2.25, *p*_fdr_ < 0.001), trimethylamine (FC = 1.42, *p*_fdr_ = 0.001) and several carnitine‐related metabolites were significantly higher in arterial plasma in HFrEF patients compared to healthy controls (*Figure* *1B*). Compared to metabolites, lipidomic analyses revealed relatively fewer significant differences between HFrEF and controls, such as greater levels of acylcarnitines like AcCa (12:0) (FC = 3.05, *p*_fdr_ < 0.001) (*Figure* *1C*). Consistent with the separation between the arterial plasma metabolomic and lipidomic profile, the plasma profile of coronary sinus blood also differed between controls and HFrEF patients (*Figure* *1D*). Comparison of the relative concentrations of metabolites in coronary sinus plasma again revealed higher levels of kynurenine (FC = 1.91, *p*_fdr_ < 0.001), 5‐HIAA (FC = 1.93, *p*_fdr_ < 0.001), carnitine‐related metabolites and xanthosine (FC = 2.10, *p*_fdr_ < 0.001), with the emergence of the ketone body acetoacetate in HFrEF (*Figure* *1E*). Lipidomic analysis demonstrated a mixed pattern in which some complex lipids were significantly lower in HFrEF coronary sinus plasma, whilst others were greater in HFrEF versus controls (*Figure* *1F*). We next identified the metabolites and lipids that were the most significantly extracted or released by the healthy and failing myocardium by determining the ratio (FC) of the coronary sinus to arterial plasma concentration. Numerous metabolites were significantly extracted in the normal heart including compounds related to energy metabolism such as creatine (FC = 0.59, *p*_fdr_ < 0.001), glutamate (FC = 0.64, *p*_fdr_ < 0.001), orotate (FC = 0.63, *p*_fdr_ = 0.006), succinate (FC = 0.61, *p*_fdr_ = 0.006), and arginosuccinate (FC = 0.57, *p*_fdr_ = 0.007). Extraction of the lipid species PC(12:0/12:0) (FC = 0.42, *p*_fdr_ = 0.04) was also evident in the normal heart. In the failing heart, extraction of creatine (FC = 0.63, *p*_fdr_ < 0.001), glutamate (FC = 0.68, *p*_fdr_ < 0.001) and orotate (FC = 0.71, *p*_fdr_ = 0.02) were again evident in HFrEF, however with the appearance of significant lactate extraction (0.78, *p*_fdr_ < 0.001) and with the loss of detectable succinate extraction. Interestingly, there was a prominent release of citraconate (citraconic acid) (FC = 1.31, *p* = 0.02, *p*_fdr_ = 0.07). Lipidomic analysis in HFrEF identified significant extraction of the lipid moieties PC(16:1/22:6) (FC = 0.56, *p*_fdr_ = 0.01), LPEt(15:0) (FC = 0.85, *p*_fdr_ = 0.02) and phSM(d38:2) (FC = 0.32, p_fdr_ = 0.02). The relative changes in the patterns of key metabolites and lipids are further depicted in *Figure* *1G,H*, as well as in online supplementary *Tables* *S2* to *S4*.

Next, we contrasted the patterns of metabolite and lipid handling in HFrEF and healthy hearts by comparing the transmyocardial FC for each compound. A switch to myocardial succinate relative release was the most significant alteration (FC = 2.02, *p*_fdr_ = 0.02). A directionally similar change in arginosuccinate turnover was also identified (FC = 1.56, *p* = 0.04, *p*_fdr_ = 0.38). Alterations in several lipids were also identified, demonstrating the conversion to the relative release of several triglyceride species. These included TG(16:1/14:0/18:2), TG(14:0/18:2/18:2), TG(16:0/18:2/18:3), all *p* < 0.001 versus healthy, with TG(16:1/14:0/18:2) *p*_fdr_ = 0.08, FC = 4.62.

### Reversibility of biochemical defects with left ventricular assist device

**Figure 2:**
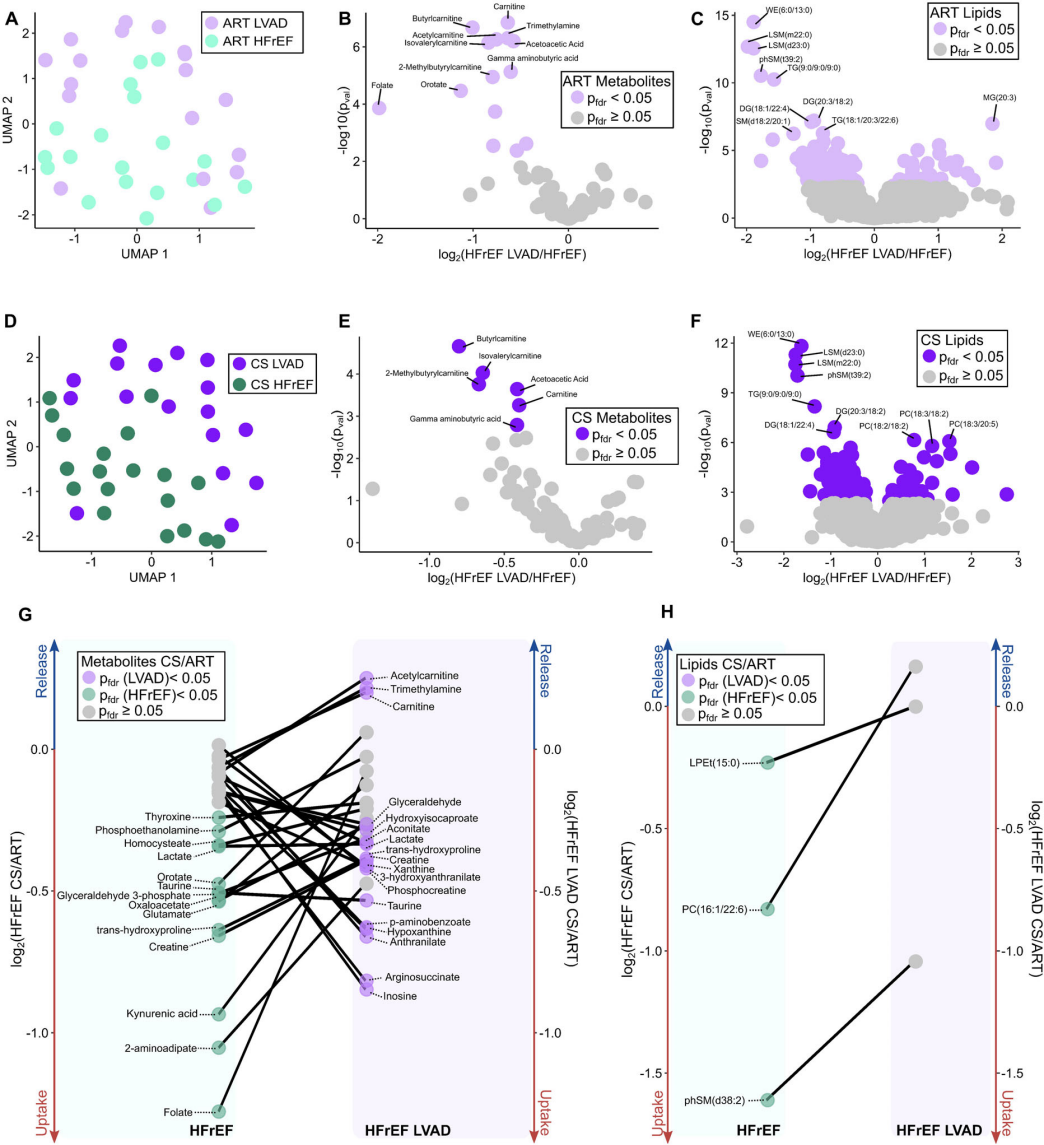
(*A*) Uniform Manifold Approximation and Projection (UMAP) of heart failure with reduced ejection fraction (HFrEF) left ventricular assist device (LVAD) and HFrEF artery samples. (*B*) Arterial (ART) metabolites compared in HFrEF LVAD and HFrEF individuals. (*C*) ART lipids compared in HFrEF LVAD and HFrEF individuals. (*D*) UMAP of HFrEF LVAD and HFrEF coronary sinus (CS) samples. (*E*) CS lipids compared in HFrEF LVAD and HFrEF individuals. (*F*) CS lipids compared in HFrEF LVAD and HFrEF individuals. (*G*) The parallel plot counterposes released/extracted metabolites (*p*_fdf_ < 0.05) in HFrEF LVAD with those in HFrEF. (*H*) The parallel plot counterposes released/extracted lipids (*p*_fdf_ < 0.05) in HFrEF LVAD with those in HFrEF.

To explore whether mechanical unloading of the failing heart was associated with reverse biochemical remodelling, we next conducted metabolomic and lipidomic profiling on arterial and coronary sinus blood samples obtained from LVAD patients. Comparison of arterial (*Figure* *2A*) and coronary sinus (*Figure* *2D*) plasma metabolomic and lipidomic profiles by UMAP scores revealed substantial separation of LVAD from HFrEF. Plasma levels of several carnitine‐related metabolites, essential for mitochondrial fatty acid oxidation, and ketone body acetoacetic acid (FC = 0.67, *p*_fdr_ < 0.001) were significantly reduced in arterial plasma in LVAD‐supported HFrEF patients compared to HFrEF patients without LVAD (*Figure* *2B*). These changes were also mirrored in the coronary sinus (*Figure* *2E*). Lipidomic analyses revealed significant reductions in the arterial levels of wax esters, WE(6:0/13:0) (FC = 0.27, *p*_fdr_ < 0.001), sphingomyelin phSM(t39:2) (FC =0.29, *p*_fdr_ < 0.001), and lyso‐sphingomyelins including LSM(m22:0) (FC = 0.25, *p*_fdr_ < 0.001) and LSM(d23:0) (FC = 0.27, *p*_fdr_ < 0.001); multiple triglycerides, such as TG(9:0/9:0/9:0) (FC = 0.33, *p*_fdr_ < 0.001); and diglycerides, including DG(20:3/18:2) (FC = 0.51, *p*_fdr_ < 0.001) (*Figure* *2C*). Many of these changes were paralleled by similar changes in coronary sinus blood (*Figure* *2F*) in LVAD‐supported HFrEF compared to HFrEF. This demonstrates that for both metabolites and lipids, cardiac uptake occurs along a concentration gradient of supply in the arterial system that substantially determines their output in the coronary sinus. *Figure* *2G* displays the release/uptake patterns of metabolites in HFrEF and LVAD hearts. It can be appreciated that, as expected, there are several overlaps. First, carnitine (FC = 1.14, *p*_fdr_ = 0.03) and acetylcarnitine (FC = 1.19, *p*_fdr_ = 0.03) were significantly released by LVAD hearts but not HFrEF hearts, suggesting they are less used by LVAD hearts or less available systemically to HFrEF hearts. The purine nucleoside inosine (FC = 0.55, *p*_fdr_ = 0.02), urea cycle intermediate arginosuccinate (FC = 0.56, *p*_fdr_ = 0.002), the aromatic acid anthranilate (FC = 0.63, *p*_fdr_ = 0.002), and the purine derivative hypoxanthine (FC = 0.64, *p*_fdr_ = 0.002) were all taken up by LVAD hearts but not HFrEF hearts (*Figure* *2G*). On the other hand, thyroxine and orotate were released by HFrEF hearts but not LVAD hearts, kynurenic acid was taken up by HFrEF hearts but not by LVAD hearts, lysine metabolite and insulin secretagogue 2‐aminoadipate was taken up by HFrEF hearts but not LVAD hearts, as was essential co‐factor folate (*Figure* *2G*). Both HFrEF and LVAD hearts retained lactate, taurine, trans‐hydroxyproline, taurine, and creatine. In addition, we investigated the relationship between the transcardiac metabolite and lipid turnover and haemodynamic parameters across the three study cohorts. Cardiac output was positively correlated with ‐log_2_FC for hydroxyproline (r = 0.48, *p*_fdr_ = 0.037) and creatine (r = 0.46, *p*_fdr_ = 0.037) indicating more extraction with higher cardiac output.

With regard to lipids, HFrEF hearts (*Figure* *2G*) retained LPEt(15:0) (FC = 0.85, *p*_fdr_ = 0.02), PC(16:1/22:6) (FC = 0.56, *p*_fdr_ = 0.01), and phSM(38:2) (FC = 0.32, *p*_fdr_ = 0.02) (*Figure* *2H*) as previously described. In contrast to HFrEF, LVAD hearts did not retain/release any complex lipids (*Figure* *2H*); however, there was a signal towards extraction of Cer(d16:0/24:1) (FC = 0.63, *p* < 0.001), TG(26:0/18:1/18:2) (FC = 0.59, *p* = 0.002); TG(16:0/18:1/20:4) (FC = 0.63, *p* = 0.03) and phosphocholine PC(18:1/22:4) (FC = 0.63, *p* = 0.004), whilst there was net release of PC(16:0/22:5) (FC =1.39, *p* = 0.003), although their p_fdr_ was above the threshold of 0.05.

### Key metabolic pathways

**Figure 3:**
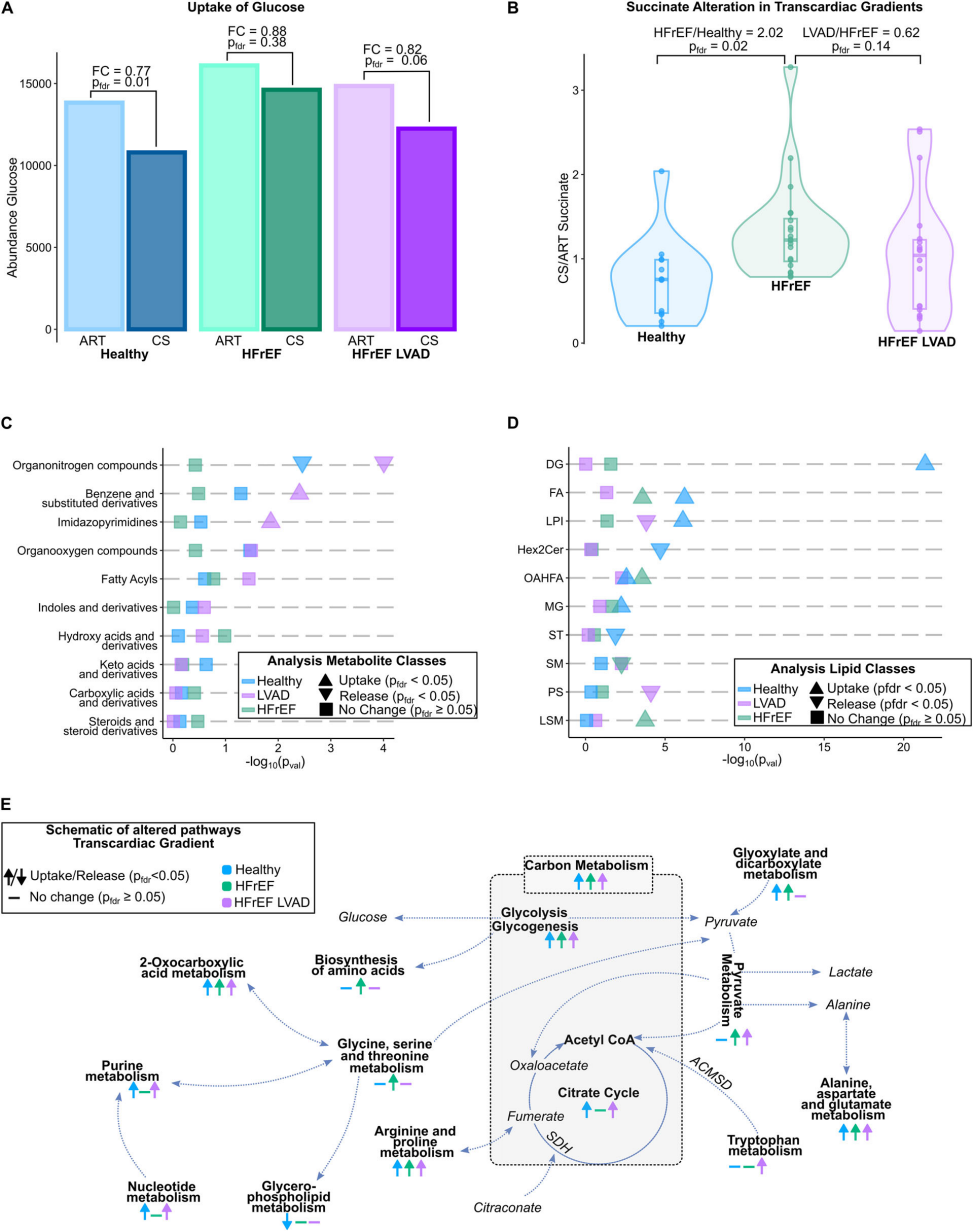
(*A*) Uptake of glucose in healthy, heart failure with reduced ejection fraction (HFrEF), and HFrEF left ventricular assist device (LVAD) cohorts. (*B*) Transcardiac succinate gradient in healthy, HFrEF, and HFrEF LVAD cohorts. (*C*) Analysis of uptake/release of metabolite classes. (*D*) Analysis of uptake/release of lipid classes. (*E*) Schematic of the altered pathways in healthy, HFrEF and HFrEF LVAD cohorts. ART, arterial blood; CS, coronary sinus; FC, fold change.

Glucose was significantly taken up by healthy hearts but not HFrEF hearts, with an almost significant restoration of glucose uptake in LVAD hearts (FC = 0.82, *p*_fdr_ = 0.06) (*Figure* *3A*). Succinate efflux was significantly greater in HFrEF compared to healthy and LVAD hearts (*Figure* *3B*). Examining other pathways, organonitrogen compounds were released by healthy and LVAD hearts but not HFrEF hearts (*Figure* *3C*). Benzene and derivatives were retained by LVAD hearts but not healthy or HFrEF hearts (*Figure* *3C*). Imidazopyridines were retained by LVAD but not healthy or HFrEF hearts (*Figure* *3C*). In terms of lipid species, diacylglycerols were avidly retained only by healthy hearts (*Figure* *3D*). Free fatty acids were extracted by HFrEF and LVAD hearts, whereas lysophosphatidylinositols were released by healthy hearts and taken up by LVAD hearts (*Figure* *3D*). Hexosamine ceramides were only released by healthy hearts, and (O‐acyl) ω‐hydroxy fatty acids (OAHFA) were extracted by healthy and HFrEF hearts (*Figure* *3D*). Monoacylglycerols were extracted only by healthy hearts, whereas sterols were released only by healthy hearts (*Figure* *3D*). Sphingomyelins were released only by HFrEF hearts, phosphatidylserines were released by LVAD hearts only. Lyso‐sphingomyelins were extracted only by HFrEF hearts (*Figure* *3D*).

As summarized in the key pathway changes (*Figure* *3E*), it can be seen that TCA intermediates were retained in healthy and LVAD hearts but not in HFrEF hearts, as were nucleotides and purines, whereas glycolytic intermediates, proline, and arginines; alanine, aspartate, and glutamate were retained by all hearts. Some pathway intermediates were only changed in healthy hearts, such as endocannabinoid uptake and glycerophospholipid release. Pyruvate was released in HFrEF and LVAD hearts, but not healthy hearts, and glycine/serine/threonine intermediates were extracted only in HFrEF hearts (*Figure* *3E*).

### Novel role for a citraconate‐related regulatory pathway in heart failure with reduced ejection fraction

**Figure 4:**
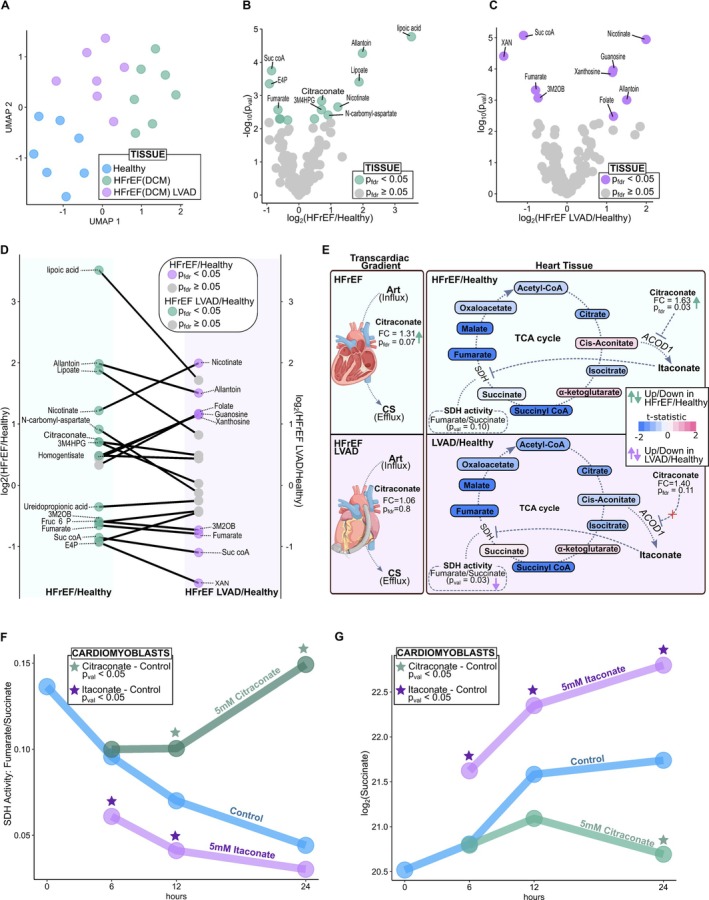
(*A*) Uniform Manifold Approximation and Projection (UMAP) of healthy, heart failure with reduced ejection fraction (HFrEF), and HFrEF left ventricular assist device (LVAD) tissue samples. (*B*) Myocardial metabolites are compared in healthy and HFrEF tissues. (*C*) Myocardial metabolites are compared in HFrEF LVAD and healthy. (*D*) The parallel plot counterposes altered metabolites (*p*_fdf_ < 0.05) in HFrEF LVAD/healthy with those in HFrEF/healthy. (*E*) Schematic of the tricarboxylic acid (TCA) cycle and citraconate pathway. The grey box connects the evidence collected in the transcardiac gradient with those in the tissue. (*F*) Succinate dehydrogenase (SDH) activity measured in cardiomyoblasts in control conditions and when exposed to citraconate and itaconate. (*G*) Succinate measured in cardiomyoblasts in control conditions and when exposed to citraconate and itaconate. Art, arterial blood; CS, coronary sinus.

To corroborate the transcardiac gradient results, we next explored the metabolite changes in human left ventricular tissue from unused healthy donor hearts, and tissue obtained at the time of transplantation from HFrEF patients and HFrEF patients with LVAD in situ. A principal component analysis plot illustrates good separation of the groups based on metabolite levels (*Figure* *4A*). As seen in *Figure* *4B*, several intermediates and products of central carbon metabolism such as TCA cycle (fumarate, succinyl‐CoA) and pentose phosphate pathway (erythrose‐4‐phosphate) were significantly decreased in HFrEF myocardium. The co‐factor lipoic acid and its conjugate base lipoate, the uric acid product allantoin, the co‐factor nicotinate (niacin), the pyrimidine intermediate n‐carbamoyl aspartate, and the norepinephrine product 3‐methoxy‐4‐hydroxyphenylglycol (3MH4PG) were all increased in HFrEF myocardium. Citraconate, which was increased in the coronary sinus efflux of HFrEF patients, was also increased in HFrEF myocardium (*Figure* *4B*). Citraconate has previously been reported to upregulate SDH enzymatic activity by competitive inhibition of itaconate^22^ in immune cells. Interestingly in HFrEF tissue, SDH activity assessed by the ratio of fumarate to succinate was not significantly different.

Myocardial tissue in LVAD also displayed a reduction in central carbon metabolites such as fumarate and succinyl‐CoA. The mitochondrial keto‐acid 3‐methyl‐2‐oxobutyrate (3M2OB) was also significantly decreased in LVAD myocardium, as was the purine metabolite and uric acid precursor, xanthine. The purine nucleoside xanthosine, on the other hand, was increased in HFrEF myocardium, suggesting inhibition of the enzyme purine nucleoside phosphorylase. Another purine nucleoside, guanosine, was increased in LVAD myocardium (*Figure* *4C*). Allantoin and nicotinate, as in HFrEF, were significantly increased in LVAD myocardium, as was the vitamin cofactor folate. Unlike HFrEF and similar to healthy myocardium, citraconate was unchanged in LVAD myocardium (*Figure* *4C*). Comparing metabolite changes in HFrEF versus healthy and LVAD versus healthy myocardium, it can be seen there was considerable overlap (*Figure* *4D*). Transcardiac release and myocardial citraconate levels returned towards control in LVAD patients (*Figure* *4E*). Therefore, we next proceeded to examine this relationship directly in cardiomyoblasts (*Figure* *4F,G*). We showed that itaconate inhibits SDH and elevates succinate levels, whereas citraconate increases SDH activity and decreases succinate levels.

## Discussion

In the current study, we comprehensively characterized myocardial metabolism using transmyocardial gradients in normal healthy control subjects, advanced HFrEF, and for the first time to our knowledge, in patients supported with LVADs, using metabolomic profiling. This approach was supported by complementary studies in ventricular tissue samples and in cardiomyoblast experiments. Consistent with prior studies, we observed a reduction in the myocardial uptake of glucose in HFrEF patients compared to controls. Intriguingly, we found a recovery of glucose uptake in LVAD hearts as opposed to non‐LVAD‐supported HFrEF hearts. Previous work also found increased glucose utilization in LVAD‐supported hearts.^23^ In this case, although glycolysis was increased in LVAD‐supported HFrEF apical myocardial tissue, it did not appear to propagate to increased oxidative phosphorylation and pyruvate oxidation through the TCA cycle.^23^ Rather, the glycolytic intermediates appeared to be directed towards the pentose‐phosphate pathway and 1‐carbon metabolism with increased utilization of glycine and serine, hypothesized to confer cardioprotection via generation of reduced nicotinamide adenine dinucleotide phosphate to enable biosynthesis and reduce oxidative stress.^23^ Our data are consistent with this prior report. First, we saw recovery of glucose uptake in LVAD‐supported hearts. Second, both glycine and serine were significantly retained by HFrEF but not HFrEF LVAD hearts, consistent with supply of glycine and serine from glycolytic intermediates. Indeed, the need to take up serine and glycine from the circulation was not evident in healthy or LVAD hearts, but only in HFrEF hearts. Third, we also saw evidence of decreased flux through the TCA cycle, and our data pointed towards inhibition of SDH as the major step mediating this impasse.

Accumulation of succinate is increasingly recognized as a mediator of ‘sterile’ metabolic inflammation,^4^, ^24^ linking disturbances in central carbon energetics with inflammation. Inflammation in HF has received renewed focus as a potential therapeutic target.^25^ A citraconate‐mediated pathway potentially linking inflammation with impaired oxidative phosphorylation was recently reported in macrophages.^22^ This work demonstrated that a metabolite of the TCA cycle intermediate cis‐aconitate, called itaconate, could inhibit SDH and lead to accumulation of succinate.^22^ Subsequent work in macrophages revealed that an itaconate isomer called citraconate could rescue the inhibition of SDH by itaconate.^26^ However, to our knowledge, we are the first to report activation of this pathway in HF, plausibly linking inflammation in HF with impaired central carbon metabolism via inhibition of SDH and accumulation of succinate. We demonstrated directly in HFrEF myocardium that citraconate levels were elevated when SDH activity was maintained; in LVAD, there was no elevation in citraconate, perhaps because it was not needed. It appears that citraconate was elevated as a compensatory mechanism in HFrEF, to mitigate itaconate's effects and rescue SDH activity as previously reported,^26^ thereby preventing accumulation of succinate. We propose that there is a ‘threshold’ effect by which citraconate is only activated to rescue SDH activity with a necessary degree of TCA cycle and other central carbon perturbation, as seen in HFrEF but not LVAD transcardiac gradients and myocardial tissue. The exact nature of signalling required to activate citraconate in this context needs to be established with further experimental work. At the pathway level, we observed that the TCA cycle was significantly down‐regulated in HFrEF hearts compared to healthy donor and LVAD hearts.

The citraconate‐SDH changes were observed in myocardium, and thus, we cannot ascribe cellular origin. It is possible that these findings related to the presence of inflammatory cells such as macrophages; however, we observed parallel findings in the transmyocardial blood sampling and left ventricular tissue components of the study. Moreover, we did demonstrate for the first time that the previously reported effects of itaconate and citraconate on SDH are mirrored in isolated cardiac cells. Therefore, these is also a possibility that the myocardial perturbations in these pathways were representative of cardiomyocytes, although further work is also needed to confirm this.

The apparent increased uptake and utilization of glucose was accompanied by an apparent increase in fatty acid oxidation in LVAD hearts, as suggested by increased acylcarnitine usage by LVAD hearts compared to HFrEF hearts. Acylcarnitines serve as the primary transporters of fatty acids into mitochondria for beta‐oxidation, and their accumulation in the context of incomplete fatty acid oxidation in HF has previously been reported.^27^ Thus, the increased excretion of unused acylcarnitines by HFrEF but not LVAD hearts may indicate impaired fatty acid oxidation in HFrEF and restoration of fatty acid oxidation in LVAD hearts. Other metabolite changes offer potential signs of healthy adaptive changes in terms of fuel use restoration after LVAD. For example, the kinetics of purines inosine and hypoxanthine were similar in healthy and LVAD hearts, but were perturbed in HFrEF hearts. Both of these purines, which are also byproducts of ATP catabolism, may indicate ongoing ischaemic injury or possibly cellular stress in HFrEF. Thyroxine was released by HFrEF hearts, but not LVAD hearts. This may indicate impaired conversion of thyroxine to triiodothyronine in HFrEF, due to impaired type 2 deiodinase. Upregulation of type 2 deiodinase in the heart was demonstrated to be protective in pre‐clinical models of HF.^28^ Compared to HFrEF hearts, LVAD hearts retained less complex lipids. This type of lipid ‘misuse’ in the failing heart is well described,^29^ and our data suggest that less lipid deposition occurs after LVAD mechanical unloading of the HFrEF heart. One of the most intriguing findings was the highly enriched organonitrogen pathway that demonstrated excretion of these compounds by the healthy and LVAD hearts but not the HFrEF heart.

Our study has several strengths and limitations. In particular, we evaluated myocardial uptake and release of metabolites and lipids in the unfasted state and in the presence of the prevailing loading state for each patient. Avoidance of fasting in studies of cardiac metabolism is crucial given the activation of glycogenolysis and stimulation of glucose uptake, which occurs in the fasted state, in parallel with an alteration in the plasma fatty acid profile. As a corollary, conclusions drawn from the analysis of tissue samples in isolation may be confounded by the effects of patient fasting prior to collection and to the potential effects of ischaemia after collection, prior to freezing. As with the majority of studies of this type, the sample size was modest, however the incorporation of transmyocardial sampling in LVAD patients provided a unique opportunity to validate findings made in the tissue samples and vice versa. The limited sample size did not allow us to specifically examine LVAD patients with clear evidence of myocardial recovery or to perform correlative analyses between loading state or contractile function and metabolic pathway activity. We did not measure SDH abundance or activity directly, rather using the ratio of fumarate to succinate as a surrogate measure as previously described.^30^

Taken together, we report several findings that validate and extend previous work, and identify a new pathway that plausibly explains the previously reported decrease in TCA cycle flux in HF. In particular, we demonstrate the potential key role in the failing heart of a novel citraconate–SDH–succinate pathway which provides new insights into the impairment in cardiac energetics. This pathway should be the focus of future mechanistic work to determine its potential as a novel therapeutic target to complement mechanical unloading or as a standalone therapy. Additionally, we report findings that support a restoration of glucose uptake/utilization and fatty acid oxidation after mechanical unloading. We also report evidence supportive of glycolytic shuttling to 1‐carbon metabolism after unloading. Additional metabolic adaptive effects appeared including restoration of thyroid signalling, and a decrease in indicators of ischaemic stress. The identification of a relative accumulation of potentially toxic organonitrogen compounds in HFrEF hearts is intriguing but warrants further investigation.

## Supporting information


**Appendix S1.** Supporting Information.
