# Supplementary material for: Mechanical unloading is accompanied by reverse metabolic remodelling in the failing heart: Identification of a novel citraconate‐mediated pathway
Source: Eur J Heart Fail. 2025 Jun 4;27(7):1342–52. doi: 10.1002/ejhf.3704 (PMC12370577; doi:10.1002/ejhf.3704)
Supplement: Supplementary file 1 — Appendix S1. Supporting Information. [file EJHF-27-1342-s001.docx]

**Supplementary Tables**

**Table 1.** *Demographic, echocardiographic and hemodynamic data.*

|  | **Controls**  **(n=13)** | **HFrEF**  **(n=20)** | **HFrEF+**  **LVAD**  **(n=18)** | **p value**  (*HFrEF vs LVAD) |
| --- | --- | --- | --- | --- |
| ***Demographics*** |  |  |  |  |
| Age (yrs) | 53 ± 2 | 55 ± 2 | 53 ± 4 | 0.53 |
| Gender (M/F) | 10/3 | 15/5 | 16/2 | 0.43 |
| BMI (kg.m^-2^) | 26 ± 1 | 25 ± 5 | 25 ± 1 | 0.62 |
| NYHA Class | - | 3 (3, 4) | 2 (2, 3) | 0.009 |
| Atrial Fibrillation, n (%) | - | 7 (35) | 9 (50) | 0.35 |
| Diabetes, n (%) | - | 4 (20) | 4 (22) | 0.77 |
| Coronary disease, n (%) | - | 8 (40) | 6 (33) | 0.67 |
| ACE/ARB/ARNI, n (%) | - | 15 (75) | 18 (100) | 0.21 |
| Beta blocker, n (%) | - | 14 (70) | 7 (39) | 0.054 |
| MRA, n (%) | - | 15 (75) | 5 (28) | 0.004 |
|  |  |  |  |  |
| ***LVAD Parameters*** |  |  |  |  |
| Pump type: HMIII | - | - | 18(100) |  |
| Pump flow (L/min) | - | - | 4.8 ± 0.2 |  |
| Support dur'n (days) | - | - | 194  (106, 292) |  |
|  |  |  |  |  |
| ***Echocardiography*** |  |  |  |  |
| LVEDD | 47 ± 2 | 68 ± 2 | 64 ± 3 | 0.30 |
| LVESD | 30 ± 1 | 59 ± 3 | 58 ± 3 | 0.93 |
| LVEF (%) | 70 ± 1 | 22 ± 2 | ^#^ |  |
| LV Mass Index (gm/m^2^) | 70 ± 11 | 143 ± 14 | 118 ± 11 | 0.16 |
|  |  |  |  |  |
| ***Hemodynamics*** |  |  |  |  |
| HR (bpm) | 62 ± 4 | 79 ± 4 | 76 ± 3 | 0.63 |
| MAP (mmHg) | 95 ± 3 | 77 ± 2 | 83 ± 2 | 0.06 |
| PCWP (mmHg) | 9 ± 1 | 22 ± 2 | 14 ± 2 | 0.005 |
| CO (L/min) | 5.2 ± 0.3 | 3.9 ± 0.3 | 5.7 ± 0.3 | <0.001 |
| LVSWI (gm.m/m^2^) | 28 ± 2 | 20 ± 2 | 6 ± 1 | <0.001 |

***Abbreviations:*** *Data are mean±SEM except for support duration (median and IQR). BMI = body mass index; ACEi = angiotensin converting enzyme inhibitor; ARB = angiotensin II receptor blocker; ARNI - angiotensin receptor blocker/neprilysin inhibitor; MRA = mineralocorticoid antagonist; HMIII - Heartmate III LVAD; LVEDD - left ventricular end diastolic dimension; LVESD - left ventricular end systolic dimension; LVEF - left ventricular ejection fraction; HR - heart rate; MAP - mean arterial pressure; PCWP - pulmonary capillary wedge pressure; CO -cardiac output; LVSWI - left ventricular stroke work index. #LVEF in LVAD patients not assessed due to imaging artefact*

***Table 2.*** *Top 5 metabolites extracted/released in healthy, HFrEF, and HFrEF+LVAD cohorts.*

| **Metabolite** | **FC** | **CI.L** | **CI.R** | **p_val_** | **p_fdr_** |
| --- | --- | --- | --- | --- | --- |
| **Healthy Transcardiac Gradient - Top 5 altered metabolites** | | | | | |
| Creatine | 0.59 | 0.50 | 0.70 | 1.41·10^-8^ | 1.59·10^-6^ |
| 4-Hydroxyproline | 0.61 | 0.51 | 0.72 | 5.17·10^-8^ | 2.92·10^-6^ |
| L-Glutamic acid | 0.64 | 0.53 | 0.77 | 7.08·10^-6^ | 2.66·10^-4^ |
| Kynurenic acid | 0.43 | 0.29 | 0.63 | 3.58 ·10^-5^ | 0.78·10^-4^ |
| Aminoadipic acid | 0.50 | 0.36 | 0.68 | 3.70 ·10^-5^ | 7.83·10^-4^ |
| **HFrEF Transcardiac Gradient - Top 5 altered metabolites** | | | | | |
| Creatine | 0.63 | 0.55 | 0.72 | 1.22 ·10^-9^ | 1.14·10^-7^ |
| trans-hydroxyproline | 0.64 | 0.56 | 0.73 | 2.03·10^-9^ | 1.14·10^-7^ |
| 2-aminoadipate | 0.48 | 0.37 | 0.62 | 1.55·10^-7^ | 5.87·10^-6^ |
| Glutamate | 0.68 | 0.59 | 0.79 | 2.49·10^-6^ | 7.04·10^-5^ |
| Lactate | 0.78 | 0.70 | 0.87 | 2.53·10^-5^ | 4.92·10^-4^ |
| **HFrEF LVAD Transcardiac Gradient - Top 5 altered metabolites** | | | | | |
| Anthranilate | 0.63 | 0.51 | 0.77 | 3.45·10^-5^ | 2.34·10^-3^ |
| p-aminobenzoate | 0.64 | 0.52 | 0.79 | 5.61·10^-5^ | 2.34·10^-3^ |
| Hypoxanthine | 0.64 | 0.52 | 0.79 | 6.21·10^-5^ | 2.34·10^-3^ |
| Lactate | 0.79 | 0.70 | 0.88 | 8.72·10^-5^ | 2.46·10^-3^ |
| Arginosuccinate | 0.56 | 0.42 | 0.75 | 1.21·10^-4^ | 2.74·10^-3^ |

FC = fold change; CI.L = 95% confidence interval, left; CI.R = 95% confidence interval, right; p_val_ = p-value; p_fdr_ = p value after adjustment for false discovery rate.

***Table 3.*** *Top 5 lipids extracted/released in the transcardiac gradient of Healthy, HFrEF, and HFrEF LVAD cohorts.*

| **Lipids** | **FC** | **CI.L** | **CI.R** | **p_val_** | **p_fdr_** |
| --- | --- | --- | --- | --- | --- |
| **Healthy Transcardiac Gradient - Top 5 altered lipids** | | | | | |
| PC(12:0/12:0) | 0.42 | 0.29 | 0.62 | 2.46·10^-5^ | 4.11·10^-2^ |
| DG(17:1/18:1) | 0.70 | 0.57 | 0.87 | 1.91·10^-3^ | 0.65 |
| OAHFA(36:1) | 0.46 | 0.28 | 0.75 | 2.01 ·10^-3^ | 0.65 |
| TG(6:0/11:2/18:3) | 0.55 | 0.37 | 0.80 | 2.12·10^-3^ | 0.65 |
| TG(14:0/18:2/18:2) | 0.45 | 0.28 | 0.74 | 2.12·10^-3^ | 0.65 |
| **HFrEF Transcardiac Gradient - Top 5 altered lipids** | | | | | |
| PC(16:1/22:6) | 0.56 | 0.44 | 0.71 | 8.96·10^-6^ | 1.49 ·10^-2^ |
| phSM(d38:2) | 0.32 | 0.19 | 0.54 | 3.51·10^-5^ | 2.31·10^-2^ |
| LPEt(15:0) | 0.85 | 0.79 | 0.91 | 4.16·10^-5^ | 2.31·10^-2^ |
| DG(18:0/18:0) | 0.84 | 0.76 | 0.92 | 3.07·10^-4^ | 0.12 |
| LPC(34:1) | 1.38 | 1.14 | 1.67 | 8.80·10^-4^ | 0.24 |
| **HFrEF LVAD Transcardiac Gradient - Top 5 altered lipids** | | | | | |
| Cer(d16:0/24:1) | 0.63 | 0.51 | 0.78 | 3.52·10^-5^ | 5.87·10^-2^ |
| TG(26:0/18:1/18:2) | 0.59 | 0.42 | 0.82 | 2.47 ·10^-3^ | 0.99 |
| TG(16:0/18:1/20:4) | 0.63 | 0.47 | 0.86 | 3.90·10^-3^ | 0.99 |
| PC(16:0/22:5) | 1.39 | 1.11 | 1.75 | 3.93·10^-3^ | 0.99 |

FC = fold change, CI.L = 95% confidence interval, left; CI.R = 95% confidence interval, right; p_val_ = p-value, p_fdr_ = p- value after adjustment for false discovery rate.

***Table 4.*** *Top 5 metabolites changed in HFrEF compared to healthy donor myocardium, and in LVAD hearts compared to healthy donor myocardium.*

| **Metabolite** | **FC** | **CI.L** | **CI.R** | **p_val_** | **p_fdr_** |
| --- | --- | --- | --- | --- | --- |
| **Tissue HFrEF/Healthy - Top 5 altered metabolites** | | | | | |
| lipoic acid | 11.41 | 4.60 | 28.28 | 1.70 ·10^-5^ | 2.16·10^-3^ |
| Allantoin | 3.95 | 2.24 | 6.98 | 5.36·10^-5^ | 3.40·10^-3^ |
| Suc CoA | 0.55 | 0.42 | 0.72 | 1.79·10^-4^ | 7.59·10^-3^ |
| Lipoate | 3.67 | 1.93 | 7.00 | 3.91·10^-4^ | 1.12·10^-2^ |
| E4P | 0.52 | 0.38 | 0.72 | 4.45·10^-4^ | 1.12·10^-2^ |
| **Tissue LVAD/Healthy - Top 5 altered metabolites** | | | | | |
| Suc CoA | 0.46 | 0.35 | 0.61 | 8.41·10^-6^ | 7.21·10^-4^ |
| Nicotinate | 3.98 | 2.40 | 6.59 | 1.13·10^-5^ | 7.21·10^-4^ |
| XAN | 0.33 | 0.21 | 0.51 | 3.88·10^-5^ | 1.64·10^-3^ |
| Guanosine | 2.23 | 1.57 | 3.18 | 1.09·10^-4^ | 3.27·10^-3^ |
| Xanthosine | 2.21 | 1.55 | 3.15 | 1.29·10^-4^ | 3.27·10^-3^ |

*Complete results are reported in Supplementary Table 6. FC = fold change; CI.L = 95% confidence interval, left; CI.R = 95% confidence interval, right; p_val_ = p-value; p_fdr_ = p-value after adjustment for false discovery rate.*
